# Adulthood trajectories of resilience and vulnerability: exploring gender differences in disadvantage after experience of out-of-home care

**DOI:** 10.1186/s12889-025-21531-y

**Published:** 2025-02-02

**Authors:** Lisa Bornscheuer, Evelina Landstedt, Karl Gauffin, Ylva B. Almquist

**Affiliations:** 1 Department of Public Health Sciences, Stockholm University, Stockholm, SE-106 91 Sweden; 2 Department of Social and Psychological Studies, Karlstads Universitet, Karlstad, Sweden

**Keywords:** Resilience, Vulnerability, Childhood adversity, Out-of-home care, Register-based research, Disadvantage, Birth cohort study

## Abstract

**Background:**

Childhood adversity places individuals in a vulnerable position, resulting in potentially enduring disadvantage across life domains like health and work. Studying the manifestation of this disadvantage is crucial for understanding which resources society can provide to mitigate or prevent it, which makes this subject a fundamental public health concern. This study investigated whether disadvantage patterns after childhood adversity differ by gender and educational level, using out-of-home care as proxy for early adversity.

**Methods:**

We used register data from a 1953 Swedish birth cohort. Distinct profiles of socioeconomic and health disadvantage in individuals with out-of-home care experience were identified using group-based multi-trajectory modelling. Multinomial logistic regression was then used to determine whether gender and education, individually or in interaction with each other, predict group membership.

**Results:**

In the population without history of out-of-home care, adulthood disadvantage was highly gendered, with women being more likely to experience disadvantage related to unemployment and poor health, while criminality and substance misuse was more common among men. History of out-of-home care was associated with a general increase in adulthood disadvantage, but the gender differences were largely absent. Women in this group were however less likely than men to experience disadvantage across multiple life domains (complex disadvantage OR = 0.56, *p* = 0.046; unemployment-related disadvantage OR = 0.51, *p* = 0.005). Higher level of education was associated with reduced likelihood of membership in the group marked by disabling health disadvantage (OR = 0.55, *p* = 0.002) and complex disadvantage (OR = 0.37, *p* = 0.001). An interaction term between gender and education was not significant.

**Conclusions:**

Adulthood disadvantage was more common in the group with history of out-of-home care. The gender differences in disadvantage present in the full cohort were largely attenuated among individuals with out-of-home care history. We showed that using administrative data on outcomes across multiple life domains can provide rich descriptions of adult experiences after childhood adversity. Future research could examine gender differences in mechanisms translating into resilient or vulnerable trajectories, including the protective potential of education in relation to specific disadvantage patterns.

**Supplementary Information:**

The online version contains supplementary material available at 10.1186/s12889-025-21531-y.

## Background

Childhood adversity (CA) is associated with numerous disadvantages across the life course. Among these are economic indicators such as unemployment [1], physical and mental ill-health [2–5], lower well-being [3], negative health behaviours [4, 6], and criminal behaviour [7]. Many of these associations follow a dose-response relationship [4, 7]. The processes that lead to or perpetuate disadvantage after adversity can be viewed as vulnerability processes [8, 9]. However, the majority of individuals exposed to CA follow life trajectories comparable to those of individuals without such experiences [10]. The absence of negative outcomes despite exposure to significant adversity is captured by the concept of resilience [8, 10, 11]. The capacity for resilience is influenced by the access to relevant resources across the life course, which in turn is shaped by social stratification, including factors such as gender and educational attainment [9, 11].

Research on CA is often limited to retrospectively collected, self-reported data, and cross-sectional analyses [5, 12, 13]. Furthermore, most studies do not explore outcomes across different life domains simultaneously, such as health and socioeconomic indicators [14]. In addition, there is still a knowledge gap regarding gender differences in adulthood outcomes after CA [11, 14, 15].

Addressing some of the issues related to data availability outlined above, our study explores longitudinal patterns of disadvantage among individuals with experience of CA in a 1953 Swedish birth cohort. We use administrative records of out-of-home care (OHC) as proxy for CA, and thereby reduce uncertainties related to retrospective self-reports. By considering outcomes across time and including different indicators across health, social, and economic life domains, we discuss resilience and vulnerability as interlinked, but dimension- or outcome-specific processes rather than mutually exclusive opposites. The focus on multiple outcome trajectories among individuals with experience of OHC allows us to explore patterns by gender and education in how resilience and vulnerability may manifest in adulthood.

As a first step, we seek to confirm previously observed associations between OHC and multi-domain adulthood disadvantage using a disadvantage sum-score, and test if this association is moderated by gender. Secondly, we focus on a subset of individuals with experience of OHC, and describe complexity in adulthood outcomes by identifying multi-domain trajectories. Lastly, we explore whether this trajectory approach can contribute to uncovering gender differences in manifestations of resilience and vulnerability. The specific research questions are as follows: (1) What associations are visible between OHC and a disadvantage sum-score in midlife in a representative population sample? (2) In what way, if any, does gender modify this association? (3) Which multi-domain outcome trajectory groups can be identified among adults with OHC experience? (4) What gender differences do we see in terms of trajectory group membership? Across all steps, we consider level of education as a factor that may intersect with gender in producing trajectories of resilience or vulnerability across different life domains.

### Childhood adversity and its impact across time and life domains

It is well established that early adversity is associated with increased risks for disadvantage in terms of health, social, and economic indicators up until mid- and old age. CA is for example associated with mental and physical ill health as well as decreased well-being [3, 12]. Similarly, CA also predicts different socioeconomic indicators, including education level and employability [16, 17], social welfare receipt [18], and criminal behaviour [7]. These associations are not only observed in isolation: CA emerges also as predictor of complex forms of disadvantage longitudinally and across different life domains, including in earlier investigations based on the same birth cohort as our study [19].

### Out-of-home care as proxy for childhood adversity

Although self-reported, often retrospective measures of CA, such as the Childhood Trauma Questionnaire (CTQ), show good psychometric properties [20], they also carry a risk of bias, such as differential recall bias [21]. One way of reducing the bias induced by retrospective self-reports is to approximate CA via records of OHC. Instances of suspected severe adversity in the family are prevalent among OHC cases, which renders OHC experience a viable proxy for different forms of CA [22, 23]. Similar to other measures of CA, OHC experience is associated with mortality [24–26], morbidity [27], and adverse socioeconomic outcomes such as unemployment [27]. In the cohort under study here, experience of OHC is, e.g., associated with all-cause mortality [22] and trajectories of social assistance, unemployment, and mental health problems [28].

### Sex and gender differences in CA exposure and impact

Sex, the biological characteristics of men and women, and gender, the corresponding socially constructed norms, roles, and behaviours, influence health and other life domains across time in interaction with each other, and their respective contributions are hard to disentangle [29]. In the following, we give some examples of how sex and gender both influence outcomes in the context of CA. Many of the patterns in these outcomes are shaped by the social construct gender rather than by biological sex (for a framework of gendered pathways to health see Heise et al. [30]). This study therefore discusses possible differences between men and women from a gender perspective.

Already the exposure to CA may vary between boys and girls, depending on the type of adversity and on the context in which it occurs. For example, many studies report that girls are more often exposed to childhood sexual abuse [31, 32], and some research indicates that boys are more often victims of physical abuse [32]. A recent study from Denmark found more complex adversity patterns in girls compared to boys [33]. Beyond differences in exposure to CA, there is evidence for more overarching sex or gender differences in the impact of CA, e.g., in terms of health outcomes. Biological sex can play a contributing role, for example through sex differences in the response to stress [34]. At the same time, also gender may shape CA’s consequences [11, 30]. The social norms and roles associated with being a man or a woman in a given context reflect and reinforce societal power structures, which in turn contribute to differences in the resources and the agency an individual has in resisting or recovering from adversity [15, 30, 35]. One example are women’s more frequent exits from and re-entries into the labour market, and a higher share of unpaid care work at home [36, 37]. This may put women in a more vulnerable position compared to men. However, gender norms can also be harmful for men, for example, men are more likely to engage in health risk behaviours when compared to women [30]. Given that gender is a structure that encompasses virtually all areas of life, we want to explore gender patterns in resilience and vulnerability in the context of CA. Evidence for differences between men and women with experience of CA can be found, e.g., in terms of specific health outcomes, such as higher rates of internalising disorders in women, even though a meta-analysis failed to produce a statistically significant pooled estimate [38]. Other examples for gender patterns in outcomes after CA can be found in relation to substance use disorders [39], or in terms of more general outcomes, such as longitudinal mortality patterns [23].

Findings on gender differences remain inconclusive also for other consequences of CA. With regard to economic indicators, some studies report that women are more severely impacted than men [1]. In terms of criminal behaviour, CA is linked to a higher relative risk for women, but in absolute terms, men are still overrepresented, both in the general population and in the CA group [40, 41]. With regards to multi-domain outcomes, Daining and DePanfilis [42] used an index measured at one timepoint and identified that women transitioning out of OHC had fewer problems than men. Similarly, previous research on the same data material as in the current study found that women less frequently experience composite patterns of disadvantage in the wake of CA than men, with mental health problems, social welfare receipt, and unemployment co-occurring [19]. Furthermore, men’s disadvantage patterns have been found to be longitudinally more stable than those among women [19]. Given these heterogeneous findings across studies, it seems likely that the observable differences between men and women depend on the context, timing of measurement, and type and operationalisation of outcome.

### Resilience and vulnerability – conceptualisation and empirical considerations

**Fig. 1:**
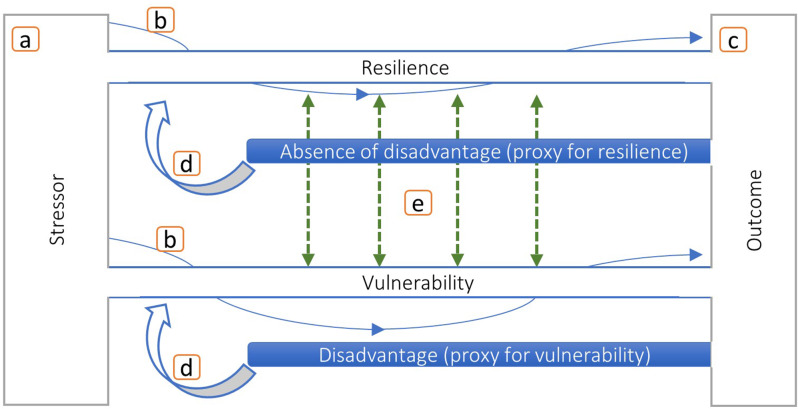
The figure illustrates resilience and vulnerability as interlinked and yet separate concepts.

CA and its consequences may be explored and understood using the concepts of resilience and vulnerability [8, 9]. Together, they form a theoretical framework for describing and understanding experiences of severe stress, as well as the heterogeneity in outcomes following from it. CA more generally, and OHC specifically, are associated with a host of disadvantages. Researchers from various fields are therefore intrigued by individuals that were exposed to CA, but that do at least “as well” as individuals from a more favourable background [43]. The process by which resources prevent disadvantage after adversity from accumulating is referred to as resilience, a concept that can be applied to any dynamic system reacting to a challenge [10, 11, 44]. Correspondingly, vulnerability is a process whereby adversity results in disadvantage or loss of functioning [8, 9]. Resilience and vulnerability play out whenever there is stress on a system (a), and the system responds to said stress in a process that can fluctuate over time (b), leading to an adaptive or maladaptive outcome (c) (Fig. 1). Within this basic framework, we can conceptualise resilience and vulnerability as domain- or even dimension-specific. This means that both can play out in parallel, and that we could witness resilience and vulnerability in the same person at the same time. This study therefore approaches resilience and vulnerability holistically, considering different markers of advantage or disadvantage simultaneously, over time, and across different outcome domains. Figure 1 illustrates the interconnectedness of resilience and vulnerability within one such outcome domain. This could be, for example, the health domain. Any individual life domain in turn comprises different dimensions. In the case of health, these could be, for example, mental and physical health. The absence of any disadvantage on one dimension can serve as proxy for resilience in that dimension, while the presence of any disadvantage can correspondingly serve as proxy for vulnerability. The repeated observation of (the absence of) disadvantage across time would then allow for a categorisation of individuals according to a combination of post-stressor trajectories across dimensions in health. Additionally, (absence of) disadvantage at one time point influences the likelihood of (absence of) disadvantage at another time point, both within (d) and across (e) dimensions of health. Furthermore, spill-over effects of resilience and vulnerability do not only occur between different dimensions of the same life domain, but also across life domains. Given that resources available to individuals are often finite, the utilisation of resources within one domain may reduce resource availability in another [9]. Moreover, ‘success’ or ‘failure’ in one domain may constitute a resource or an additional stressor in another. This is in line with ideas concerning the multidimensionality of the life course [9], and a conceptualisation of resilience as multisystemic [11, 44]. For example, we could imagine an individual that after an adverse event maintains or builds resilience in the social domain, e.g., maintaining a strong network of friends, while becoming increasingly vulnerable in the economic domain, e.g., being employed in a series of precarious job positions.

In empirical studies, resilience has been operationalised in terms of various outcomes: as the absence of illness, for example in terms of depression or anxiety [45], as composite measure combining different life domains [42], or via validated scales such as the Connor-Davidson-Resilience-Scale [46]. Vulnerability can correspondingly be operationalised in terms of the presence of illness or other forms of disadvantage. Furthermore, conceptualising resilience and vulnerability as processes rather than static requires studies which rely on longitudinal outcome measurements [11, 47]. Given the heterogeneity in operationalisations of resilience and vulnerability, and the mixed findings on sex or gender differences, contextualisation is key: resilient or vulnerable *to what*, after *what kind of exposure*, in *what kind of setting*.

## Education as an early marker and enabler of resilience

It is important to note that gender is but one stratifying principle of society, and intersects with many others [48]. For example, socioeconomic status (SES), and level of education in particular, may reflect resources that an individual can access in order to recover from adversity [30]. Although the SES-gender intersection is not the primary focus of the present study, we acknowledge the plausible influence of SES for processes of resilience and vulnerability. Educational level is a relatively stable marker of social position, and is associated with decreased risks of numerous disease outcomes and better labour market outcomes [49, 50]. In the context of OHC, higher educational levels can thus be seen as having a dual function – they can be both viewed as an early marker of resilience, and as an enabler of resilience moving forward. In the European context, women are overrepresented in higher education [51], and may therefore benefit more often than men from education in resilience processes.

## Context - the Swedish Welfare State

As indicated above, resilience and vulnerability are context-dependent in multiple ways. Firstly, what is adaptive and conducive to functioning in one setting may be considered maladaptive in another. Secondly, ways in which vulnerability and resilience may manifest are delineated by the structures within which they unfold. Thirdly, the adversity that may set these processes off is shaped by the wider circumstances. Below, we therefore provide a brief overview of the context in which our birth cohort grew up and aged, namely the greater Stockholm metropolitan area in the 1950s and 1960s, with the outcomes being measured in midlife, i.e., in the 2000s and 2010s.

### Child welfare services in the 1950s and 1960s

Until the late 1970s, Sweden had higher rates of children being taken into OHC than other comparable countries [52]. At the time, the guiding principle of the child welfare system was child protection. Just as today, placement was arranged when a child’s health and safety was deemed to be in danger either due to own behaviour or due to family circumstances. In the early days of the Swedish child welfare system, this meant that household dysfunction was viewed as leaving authorities with no choice but to intervene [53]. Only after 1980 was there a change towards accommodating families’ needs more holistically: The Swedish child welfare system transitioned from a heavy emphasis on child protection as a unilateral endeavour to the provision of support on a voluntary basis where possible, with family preservation as guiding principle [52, 53].

## Social welfare in the 2000s

Universalism and the de-commodification of social rights represent the underpinning core ideas of the Swedish welfare state [53]. In recent decades, there has been a roll-back of public expenditure and an increase of privatisation in numerous sectors, but healthcare provision remains largely free [54]. Accordingly, healthcare seeking behaviour is less restricted by own income and possibly a more accurate representation of the population’s health needs than in contexts with a strong reliance on out-of-pocket payments. Furthermore, the state provides unemployment benefits via voluntary membership in unemployment insurance funds [55], and means-tested and health-status related benefits, such as social assistance [54] and early retirement benefits [56]. The exact eligibility criteria for and conditions linked to the administration and receipt of the different benefits have changed over the past years, usually becoming more restrictive [54, 57], but overall, Swedish welfare policy can still be described as relatively universal [57].

## Gender and the Swedish Welfare State

Sweden is known for its relative gender equality, and the Swedish welfare state has actively tried to reduce gender inequalities since the 1920s and 1930s [37]. Among the policy measures taken were anti-discrimination laws to safeguard women’s right to work, changes in taxation – from joint to individual taxation of married couples – and the gradual expansion of parental leave, including earmarked parental leave months for mothers and fathers [37]. These policy measures have often met broad societal and political consensus, and most have been implemented throughout the 1970s, around a time where the study cohort was transitioning into adulthood [37]. This means that more women in this cohort were working compared to previous generations. However, gendered patterns of paid and unpaid work persist also in Sweden, and a lower proportion of women than men are part of the work force [37]. Furthermore, there continues to be a labour market segregation in terms of gender, with women, e.g., doing a greater share of part-time paid work and unpaid care work than men [37].

### Methods

#### Data material and study sample

This study uses linked register data from the Stockholm Birth Cohort Multigenerational Study (SBC Multigen), which is comprised of all individuals born in 1953 and living in the greater metropolitan area of Stockholm in 1963 (*n* = 14 608) [58]. Our full study sample included those 13,692 cohort members that were alive in 2001 and registered in Sweden for at least one year during the 15 years of follow-up (2001–2015). A flow-chart of the study sample is available in the supplementary material (Figure S1).

### Variables

*Out-of-home care experience*: Information on OHC experience was obtained from the municipal Social Registers. OHC was operationalised as binary variable, with any placement in residential or foster family care due to family circumstances (ages 0–19, 1953–1972) coded as 1, and no placements and placements exclusively due to own behaviour coded as 0. This was done in order to ensure that OHC served as viable proxy for CA.

*Gender*: Sex registered at birth was used as proxy for gender. It was obtained from the Total Population Register (TPR), and operationalised as binary variable (man/woman).

*Level of education*: Highest level of education was measured at age 37 and operationalised as binary variable, distinguishing between compulsory or upper secondary and higher education (0/1). The information was retrieved from the (Longitudinal Integrated Database for Health Insurance and Labour Market Studies) LISA register. We chose age 37 for measurement of level of education, because this was the earliest datapoint available, and the majority of cohort members had finalised their educational careers at this point.

*Social*,* economic*,* and health-related disadvantage in adulthood*: We used the Swedish National Patient Register (NPR), the LISA register, and the National Crime Register to derive six binary indicators of adulthood outcomes (ages 48–62, 2001–2015) across different life domains, both individually and in form of a disadvantage sum-score. The individual dimensions covered by the indicators are located at the intersections of social, economic, and health-related life domains (Figure S2).

*Unemployment*: Days in unemployment were retrieved from the LISA register. The indicator was coded 1 for 30 or more days of unemployment in a given year, and 0 otherwise.

*Social assistance*: Any amount of social assistance received during a given year was coded as 1, whereas absence of any registered amount of social welfare in LISA was coded as 0. Social assistance is a means tested financial support for individuals and families who live below a certain income threshold.

*Disability pension*: Information on disability pension was also obtained from LISA, with any amount of pension received in this category resulting in the observation being coded as 1, and else as 0. Disability pension can be applied for when there is an at least one fourth reduction in the ability to work for work-relevant health reasons [59].

*Affective disorders (AD) and self-harm*: Information was retrieved from the NPR, which contains both in- and outpatient care. Included were primary and secondary diagnoses related to suicide attempts, self-harm, several anxiety-related disorders such as post-traumatic stress disorder, as well as depression-related diagnoses. For each year, having received at least one of the diagnoses included in this category was coded as 1, and 0 otherwise. We considered AD and self-harm to both be forms of or related to internalising problems that are usually more common in women [60, 61].

*Substance use and crime*: This indicator includes information on alcohol and drug use related diagnoses, retrieved from the NPR, and any kind of criminal offence as registered in the National Crime Register. For each year, having experienced at least one event or diagnosis included in this category was coded as 1, and 0 otherwise. We considered substance use and crime to both be forms of or related to externalising problems that are usually more common in men [62–64].

*Somatic morbidity*: The somatic disease indicator included all diagnoses of cancers, metabolic, circulatory, and respiratory disorders recorded in the NPR. For each year, having received at least one of the diagnoses included in this category was coded as 1, and 0 otherwise. *Disadvantage sum-score*: The six socioeconomic and health indicators outlined above were summarised in a count variable. The count ranged from 0 – no event in any of the years in any of the dimensions (no disadvantage) – to 5, i.e., events in at least 5 of the 6 dimensions when considering the entire follow-up time (complex disadvantage). Due to the time span of our follow-up, all diagnoses were made according to ICD version 10. The full list of diagnostic codes included in each indicator is available in the Appendix (Table S1).

### Statistical analysis

As a first step, we provide descriptive statistics by OHC experience and gender, including p-values from Pearson’s chi squared tests to test for gender differences in indicator prevalence within the two OHC categories. We then conducted ordinal regression with the disadvantage sum-score as outcome as complete case analysis (*n* = 12,586), in order to confirm that OHC experience is associated with disadvantage in adulthood in our cohort. We added gender and an interaction term between gender and OHC to the model, to assess whether there is evidence for gender being a moderator of OHC. We furthermore added educational level as a covariate.

In a second step, we approached resilience and vulnerability through health and socioeconomic trajectories in adults with experience of OHC using group-based multi-trajectory modelling (GBMTM). GBMTM is an extension of group-based trajectory modelling, and allows observations to cluster according to trajectories of multiple indicators simultaneously, rather than accommodating just one outcome [65, 66]. GBMTM was conducted as year-wise complete case analysis, with observations with missing information for a given year being dropped from that year, but remaining in the overall GBMTM sample. Restricting the full sample to individuals with experience of OHC resulted in a sub-sample of size *n* = 929. The best fitting clustering around trajectory groups was selected using Akaike Information Criterion (AIC) and Bayesian Information Criterion (BIC), which are relative measures used for the comparison of alternative models, with the value closest to 0 indicating the best fit. In addition, we considered entropy, with values closer to 1 indicating a better fit, and substantive interest, or “usefulness” of the model output in relation to the research question [65]. From the GBMTM output, we created a group membership variable, with each trajectory group being one outcome category. Using this group membership variable as the outcome, we conducted multinomial regression and plotted the resulting predicted marginal probabilities of group membership by gender and level of education. Data is censored for death and migration (please see Tables S2a and S2b for further information). All statistical analyses were conducted in Stata version 18.5.

### Ethics

The Regional Ethical Review Board in Stockholm approved the creation of SBC Multigen (no. 2017/34 − 31/5; 2017/684 − 32). The research project Risk and resilience: Pathways to (ill)health among men and women with experiences of childhood adversity (RISE), of which this study is part, is covered under ethical approval no. 2019–04376.

## Results

### Associations between experience of OHC, gender, and socioeconomic and health indicators

The prevalence of each indicator, across the whole follow-up, by experience of OHC and gender is presented in Table 1 (based on the full sample, *n* = 13,692). All disadvantage indicators are more prevalent in the OHC than in the non-OHC group. Prevalence of OHC experience due to family reasons was relatively evenly distributed between men and women in this cohort, including length of placement and type of placement (see Table S3a-S3b for frequency distributions across OHC characteristics). Gender differences were observable for a number of disadvantage indicators in those without OHC experience, but only in terms of substance use and crime in those with OHC experience. Similarly, there was a statistically significant difference in distribution of gender across the disadvantage sum-score categories only in those without experience of OHC (*p* = 0.007), but not in those with such experience (*p* = 0.57) (Table S4). A higher proportion of women than men in both the OHC and no OHC experience group attended upper secondary or higher education.

**Table 1:** Frequency and prevalence (n (%)) of disadvantage indicators (ever vs. never experienced during follow-up) and education by experience of OHC and gender

	No OHC experience					OHC experience				
	Men		Women		p-value	Men		Women		p-value
	n	%	n	%		N	%	n	%	
Unemployment	1,487	23.0	1,328	21.1	0.012	147	30.4	119	26.7	0.22
Social welfare receipt	667	10.3	638	10.1	0.77	108	22.3	103	23.1	0.76
Disability pension	864	13.3	1,247	19.8	< 0.001	128	26.4	129	29.0	0.39
Affective disorders & Self-harm	431	6.7	563	9.0	< 0.001	57	11.8	70	15.7	0.08
Substance use & Crime	928	14.3	383	6.1	< 0.001	114	23.6	56	12.6	< 0.001
Somatic morbidity	3,343	51.6	3,361	53.5	0.039	256	52.9	252	56.6	0.25
Education	5,032	78.6	5,304	85.2	< 0.001	317	66.3	321	73.1	0.025
Total	6,475		6,288			484		445		

* total *n* = 13,692; p-values derived from Pearson’s chi-squared tests for within OHC group comparisons between male and female for each indicator; education: highest educational level upper secondary or higher

The ordinal regression of OHC experience on disadvantage sum-score showed an estimated 81% increase in odds of a one-category increase in the score when comparing those with OHC experience to those without (95% CI 1.59–2.07; *p* < 0.001) (Table S5). Adding gender to the model resulted in a nearly unchanged estimate for OHC experience. Being a woman was associated with an about 10% increase in odds of a higher disadvantage score compared to men in individuals with the same OHC experience status (95% CI 1.03–1.17, *p* = 0.004). Adding educational level resulted in a slightly lower estimate for OHC experience (OR 1.70; 95% CI 1.49, 1.94; *p* < 0.001) and a slightly higher estimate for gender (OR 1.14; 95% CI 1.07, 1.21; *p* < 0.001). Having upper secondary or higher education is associated with a decrease in odds of being in the next higher disadvantage sum score category (OR 0.57; 95% CI 0.52, 0.62; *p* < 0.001). The interaction term between gender and OHC was not statistically significant.

### Multidomain trajectories among cohort members with experience of OHC

**Fig. 2:**
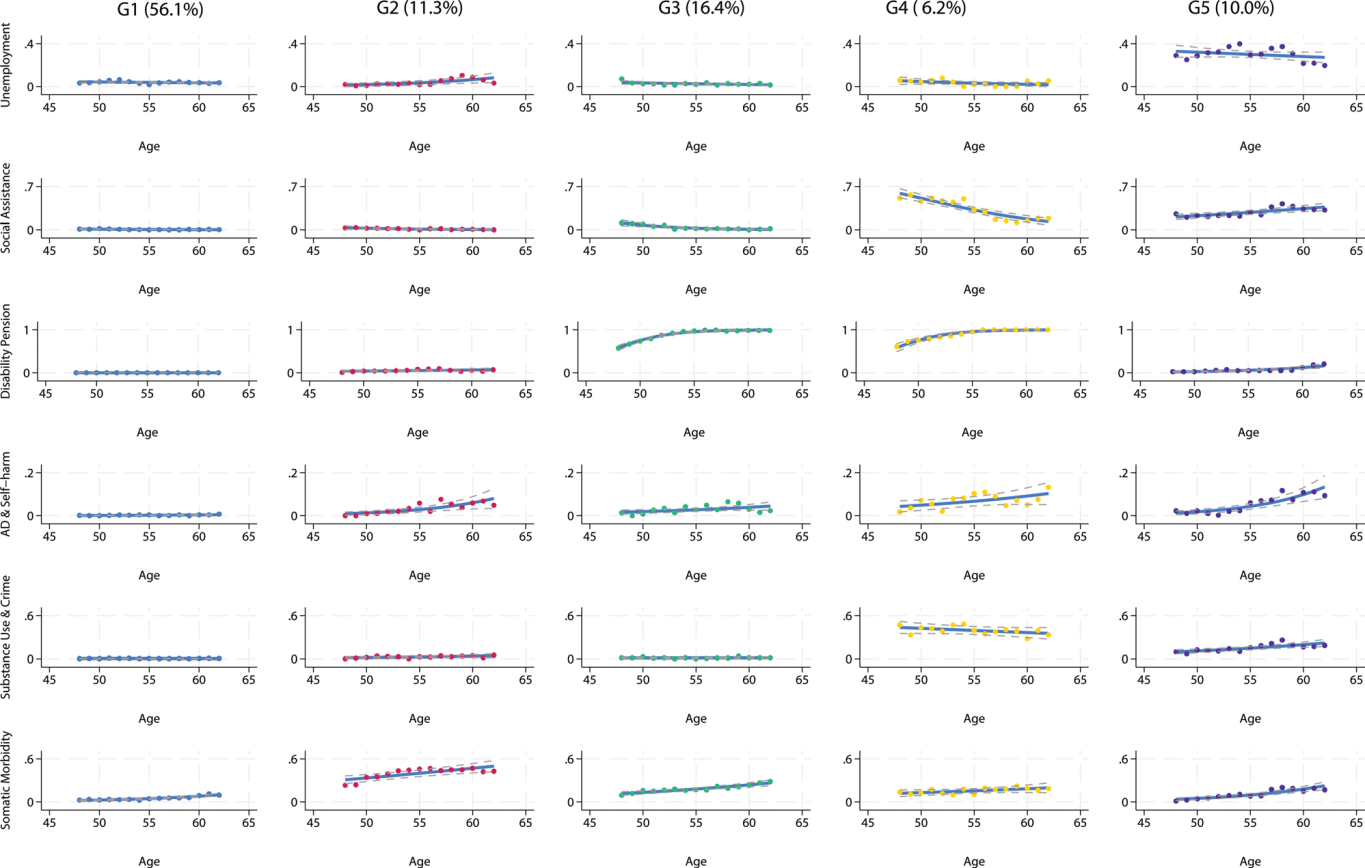
GBMT results in cohort members with experience of OHC due to family reasons based on 929 individuals.

For the GBMTM, we considered the five-group linear polynomial model to have the best fit overall. An overview of the model fit statistics for all models is available in the supplementary material (Table S6), as well as group membership probabilities for the chosen model (Table S7). The five trajectory groups from the best fitting model in adults with OHC experience were plotted in terms of the respective group-level event probabilities (Fig. 2). To describe the discerning characteristics of each trajectory group, we focussed on the number of dimensions with increased event probabilities, and the relative magnitude of these probabilities.

The biggest group (G1), which had been assigned 56.1% of observations, was the group with the lowest probabilities of experiencing any outcome. G2, which comprised 11.3% of individuals in this subsample, had comparatively low event probabilities for most indicators, but high probabilities on the somatic morbidity indicator, as well as elevated probabilities of AD and self-harm. On the AD and self-harm dimension, probability of experiencing an outcome increased from about age 55, and on the somatic morbidity indicator from around age 50. G3 (16.4%) was also characterised by health problems, which gradually increased with age, but here, the dominant indicator was receipt of disability pension, with all group members having left the labour market before age 55. G4 (6.2%) and G5 (10.0%) were groups with elevated probabilities of experiencing an event in most or all dimensions. The dominant indicators for G4 and G5 were disability pension and unemployment, respectively. All individuals allocated to G4 had left the labour market between age 55 and 60. There were initially high levels of social assistance that slightly decreased with time, possibly because social assistance in this group was gradually substituted by early retirement benefits. Substance use and crime probability decreased slightly with time, while AD and self-harm probability slightly increased. In G5, most indicators had elevated probabilities, and increasing slightly with time. Unemployment decreased over time, which is to be expected, as individuals begin to exit the labour market with increasing age. Notably, there was a relatively steep increase in AD and self-harm from around age 55 on in this group.

### Associations with gender and education

The trajectory groups described above served as categories of the dependent variable in the subsequent multinomial regression analysis. Observation count and percentage for each of the groups, as well as an overview of trajectory labels and brief descriptions of the main group characteristics, are displayed in Table 2.

**Table 2:** Trajectory group labels and descriptions

Trajectory Group	Label	Dimensionality of disadvantage	Main characteristics	*n* (%)
G1	No disadvantage	n/a	Low event probabilities	527 (56.1)
G2	Health disadvantage	Bidimensional	Somatic morbidity dominant dimension; some events in AD & Self-harm	100 (11.3)
G3	Disabling health disadvantage	Bidimensional	Health problems resulting in receipt of disability pension	154 (16.4)
G4	Complex disadvantage	Multidimensional	Events across all indicators except unemployment	57 (6.2)
G5	Unemployment-related disadvantage	Multidimensional	Unemployment dominant domain; events across all indicators, mostly increasing with age	91 (10.0)
Total				929

**Fig. 3:**
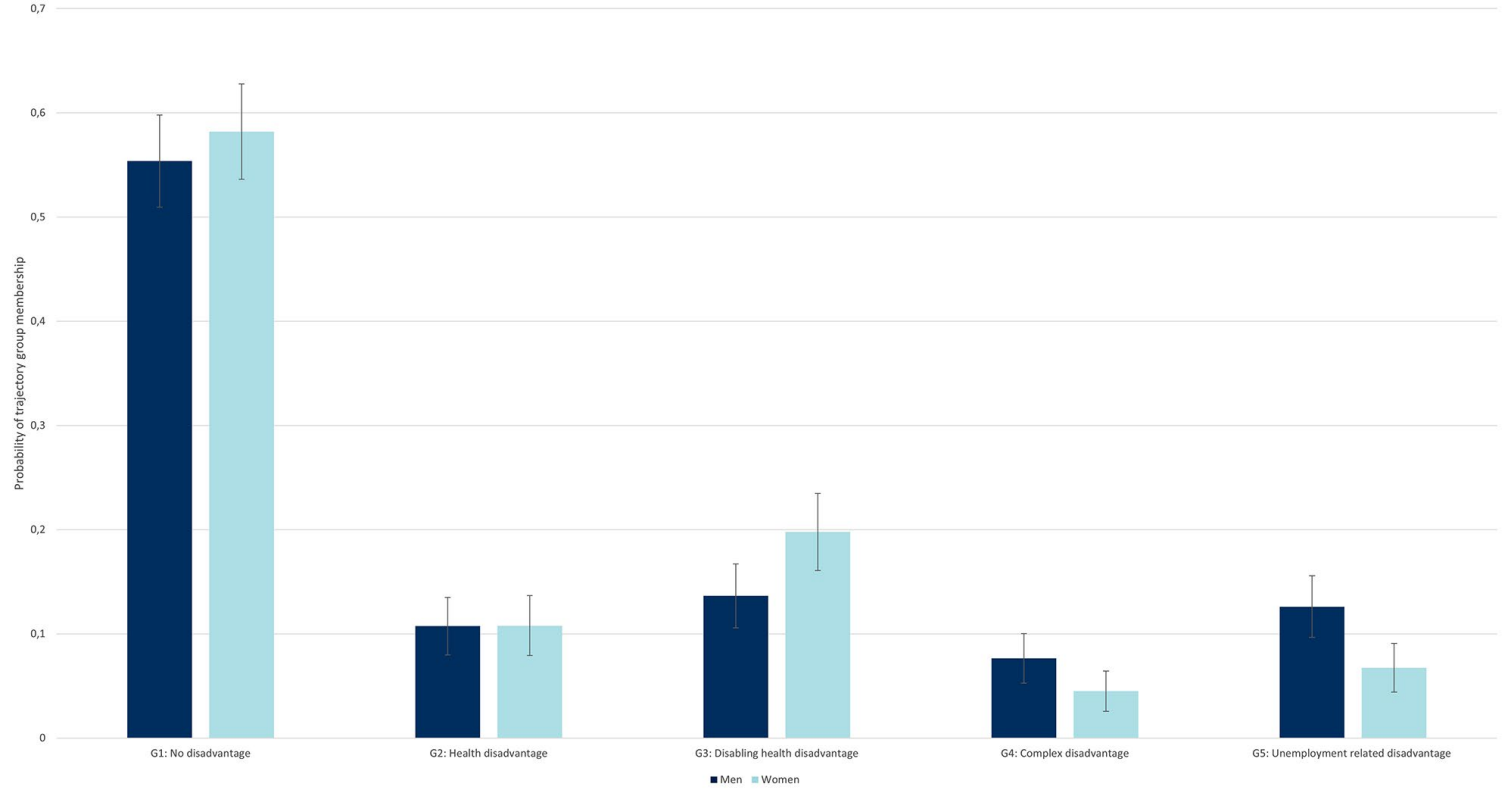
Plot of predicted probabilities of group membership by gender, not adjusted for education

According to the multinomial regression analysis of gender and level of education on trajectory group membership, women were less likely than men to follow multidomain trajectories characterised by complex disadvantage (G4; OR = 0.56, *p* = 0.046) and unemployment-related disadvantage (G5; OR = 0.51, *p* = 0.005), compared to the trajectory of no disadvantage. Some gender patterns were also observed for the other trajectory groups; however, none of these reached a statistically significant level (full results in Table S8). Predicted probabilities are presented in Fig. 3; a corresponding figure for education is available in the supplementary material (Figure S3). There was a slight tendency for women to be overrepresented in G3 (disabling health disadvantage). Adding education as covariate to the model, gender became a significant predictor of membership in G3 (OR 1.52, *p* = 0.027), but no longer of G4 (OR = 0.61, *p* = 0.099). The estimate for the association of gender and membership in G5 hardly changed when introducing education. Higher level of education was associated with reduced likelihood of membership in G3 and G4 (OR = 0.55, *p* = 0.002; OR = 0.37, *p* = 0.001). An interaction term between gender and level of education was not statistically significant.

## Discussion

In line with previous research, we found OHC experience to be associated with increased odds of disadvantage in adulthood, while higher educational level was protective. Gender did not moderate the association between OHC and disadvantage measures. In individuals with OHC experience, gender differences in disadvantage indicators that could be observed in unexposed individuals were largely not detectable. In that sense, one could see CA as an overriding experience that possibly cancels out any significant life advantage that men might otherwise have over women in terms of, for example, labour market participation [67].

By focusing on individuals with experience of OHC, and decomposing disadvantage from a crude score to multi-dimensional trajectories, we then engaged more closely with gender patterns in relation to resilience and vulnerability after CA. The analyses of multi-dimensional disadvantage trajectories across socioeconomic and health domains showed that men are overrepresented in the two groups with disadvantage in multiple dimensions across domains, G4 (complex disadvantage) and G5 (unemployment-related disadvantage). These are the groups with highest probabilities of both AD and self-harm, as well as substance use and criminal behaviour. In general population samples, we often see a stronger representation of women when it comes to AD and self-harm [60]. In this sub-sample of individuals with experience of OHC, this does not seem to be the case to the same extent. However, there is a clear gender pattern in regard to substance use and criminal behaviour; a greater relative importance of this disadvantage indicator for distinguishing the multi-dimensional disadvantage groups from other groups may be one explanation for men being over-represented in these.

In addition to not following the “typical” gender pattern in terms of prevalence of internalising problems, women with OHC experience seem to more often than men experience disadvantage that is limited to one or two dimensions. Women are however overrepresented in G3, the group characterised by disabling health disadvantage. In that sense, one could tentatively argue that once vulnerability manifests for men, it is likely to spill over into other life domains, while in women, vulnerability is more often contained in one domain of life, maintaining resilience in others - with the exception of where health disadvantage translates into an inability to work. Higher educational level is protective of at least some disadvantage patterns. It was not protective for one of the complex disadvantage groups (G5). This could mean that this specific disadvantage pattern is harder to counteract via the resources provided by education, or that men, who were overrepresented in this trajectory group, do not benefit from a higher level of education despite OHC experience to the same extent as women do. As is the case for many other studies on resilience, the majority of individuals with OHC experience are in the trajectory group without elevated risk levels in any of the disadvantage domains. It would be worth exploring in cross-national comparisons how much the “resilience prevalence” after comparable exposures and using similar resilience indicators varies between countries, to better understand the gains in resilience that a welfare state system can afford its citizens.

Our findings add nuance to previous studies on patterns of disadvantage in this cohort, where similar patterns were observed [19].We used a different method and slightly different follow-up period, and focused on individuals that experienced OHC due to family-related problems in order to specifically discuss disadvantage within a resilience and vulnerability framework. Furthermore, we added a more varied set of outcome indicators, which captures a wider range of different disadvantages. This increased the chances of describing differences in patterns between men and women in greater detail, and allowed us to approach resilience and vulnerability empirically as fluid and dimension-specific (Fig. 1).

Taken together, what we observe in this study may mean that experience of OHC – which we here assume to reflect an underlying experience of CA – levels down the playing field, and evens out parts of otherwise observable gender differences: In the face of adversity, men and women may be more alike in terms of outcome trajectories than would be expected in the absence of it. However, some differences remain, and additionally, it is important to remember that any similarity in outcome trajectories does not mean that the mechanisms leading to these outcomes are the same for men and women. Therefore, research that investigates mediators and moderators of adversity from a gender sensitive perspective is needed to describe resilience and vulnerability in this context in further detail, going beyond a description of (intermediate) outcomes, as this study has done. Our study provided some evidence that education may be a protective factor in the context of OHC experience, and possibly one that women can benefit more often from than men.

### Strengths and limitations

The study has several strengths. For example, we used prospectively collected register data, including for the approximation of CA. We furthermore allowed for a complex conceptualisation of resilience and vulnerability via multiple indicators of disadvantage, measured over a relatively long time period. This holistic, longitudinal approach to resilience and vulnerability via register data is new. Furthermore, the sample was relatively large, and we should therefore be able to identify even smaller gender differences. However, additional insights could be gained by adding, for example, more qualitative measures such as subjective indicators of well-being. This would enable a conceptualisation of resilience as something more than the absence of disadvantage, in line with a conceptualisation of health as being more than the absence of disease, and allow for a more context-sensitive understanding of resilience and vulnerability [47, 68]. Similarly, future studies, and especially those that aim at causal inference in the context of acute adverse events, would gain by including a measure of pre-adversity functioning [47].

Despite access to high quality data, there is always risk of bias in estimating associations. Due to the set-up of the birth cohort, people migrating from the Stockholm metropolitan area before their 10th birthday are not included. It does not seem likely that this would impact representativeness of the group of OHC-exposed individuals in this study, as we are not aware of conclusive evidence for differential migration patterns from metropolitan areas by CA experience. Furthermore, in order to reduce overestimation of resilient outcomes, we adjusted for death and migration. Another caveat is health selection, with the most afflicted cohort members dying before the beginning of follow-up [23]. This is likely to lead to underestimation of the prevalence of all disadvantage indicators.

Using register data also comes with some limitations. Choosing OHC records as proxy for CA implies that we are looking at relatively severe cases. This may negatively impact generalisability to less severe forms of CA, and even more so in as far as OHC experience can be an adversity in its own right [27, 69]. The underlying assumption of our study is that OHC is a measure taken in the best interest of the child and with the potential to ease the burden on child and family, but we fully acknowledge that this is not always the case and that in some instances, OHC can be an exacerbating factor [27, 69]. Furthermore, it was beyond the scope of the current study to investigate events occurring between the experience of OHC and the beginning of follow-up in adulthood. Since the aim was not to determine mechanisms linking adversity and later disadvantage, this does not invalidate our descriptive engagement with longitudinal outcome patterns. However, given that registers capture severe forms of disadvantage, it is likely that the size of the no disadvantage trajectory group is overestimated. At the same time, it is unlikely that this misclassification would vary by gender. Hence, while the group size may be incorrect, this is unlikely to introduce bias in terms of gender patterns. Lastly, it is possible that those gender patterns that we observe in outcome trajectories are in fact due to, or at least co-created by, gender patterns in the exposure. For example, we could imagine that social workers treat girls and boys differently. This is something we cannot capture in our study, but since there are no gender differences in placement length or other placement characteristics, and since we only focused on placements due to family circumstances rather than own behaviour, this seems unlikely. Lastly, our focus on heterogeneity in the outcome meant we neglected heterogeneity in the exposure, which is of interest, too, for it may play a role in type and severity of outcomes observed [70, 71]. The choice to not delve deeper into the different types of adversity was pragmatic, partly to ensure a large enough sample for multiple indicators while stratifying by gender and education, partly due to lack of more fine-grained information on the exposure, and partly in order to have one complexity rather than two to discuss. This is an important caveat to bear in mind when interpreting the findings, and Tables S3a and S3b provide descriptive statistics on those characteristics of placements that we could access. However, with previous studies having confirmed the suitability of using OHC as proxy for CA [22], we are confident that OHC was fit for purpose in the context of our study.

There are some minor issues with the methodology and conceptualisation within this study that need to be taken into consideration when interpreting the findings. Firstly, indicators of disadvantage may have been constructed in a way that hides some of the most striking differences between men and women. To avoid this, most indicators covered only one dimension, e.g., unemployment. However, some comprised multiple dimensions across several domains. In this case, we took care to not collapse “typically female” and “typically male” disadvantage into one indicator. Future studies could benefit from considering additional outcomes. A lack of suitable indicators may also be the reason for the relative lack of observable gender patterns in outcome trajectories, but since we chose proxies for socioeconomic wellbeing and health that usually follow a gender pattern, this seems unlikely. Lastly, Sweden is a relatively gender equal context compared to other countries [37], and the context in which our cohort grew up and aged was characterised by a strong social welfare system, despite recent cut-backs [54, 57, 72]. This means that the observed patterns are probably not directly translatable to contexts outside of the Nordic countries, or even to other generations within Sweden.

## Conclusions

In conclusion, this study provides a detailed description of the main profiles of adulthood disadvantage in a population exposed to adversity in childhood. As in most comparable studies, the majority of the population does not suffer from lasting observable consequences from the experiences of this adversity. We then exploited the richness of our data to explore gender differences in disadvantage patterns after CA, thus showing how routine data can be harnessed to empirically approach resilience and vulnerability in this context. Even though gender difference became less pronounced in the presence of adversity, it is important to raise awareness about resilience and vulnerability possibly manifesting in multiple ways. Given that this research is set in Sweden, our study provides some evidence that even in relatively gender equal settings, vulnerability and resilience remain gendered to a certain extent. This speaks to the overarching value of addressing gender inequalities on societal level. There was a tendency of men to experience more complex disadvantage, which may be interpreted as greater spill-over effects of vulnerability once resilience has been eroded in one life domain than is the case for women. Conducting similar studies with data from different settings would be beneficial for furthering our understanding of the contribution of gender to manifestations of resilience and vulnerability in men and women. In addition, studies are needed that provide insights into possible gender differences in the pathways along which resilience and vulnerability in the context of CA unfold.

## Electronic supplementary material

Below is the link to the electronic supplementary material.

Supplementary Material 1

## Data Availability

The datasets generated and/or analysed during the current study are not publicly available due to lacking ethical approval for data sharing. Interested researchers are encouraged to directly apply to the registry holders in Sweden.

## Abbreviations

AD: Affective Disorders
AIC: Akaike Information Criterion
BIC: Bayesian Information Criterion
CA: Childhood Adversity
GBMTM: Group–based multi–trajectory modelling
ICD: International Classification of Diseases
LISA: Longitudinal Integrated Database for health insurance and labour market studies
NPR: National Patient Register
OHC: Out–of–home Care
SBC: Stockholm Birth Cohort
SES: Socioeconomic Status
TPR: Total Population Register
