# Supplementary material for: Adulthood trajectories of resilience and vulnerability: exploring gender differences in disadvantage after experience of out-of-home care
Source: BMC Public Health. 2025 Feb 2;25:417. doi: 10.1186/s12889-025-21531-y (PMC11789295; doi:10.1186/s12889-025-21531-y)
Supplement: Supplementary file 1 — Supplementary Material 1 [file 12889_2025_21531_MOESM1_ESM.docx]

**Supplementary material**


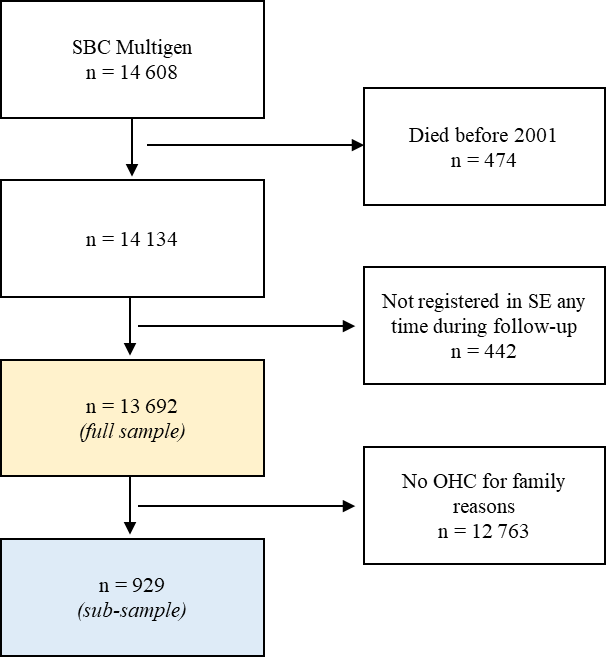


Figure S1: Flow-chart of the study sample


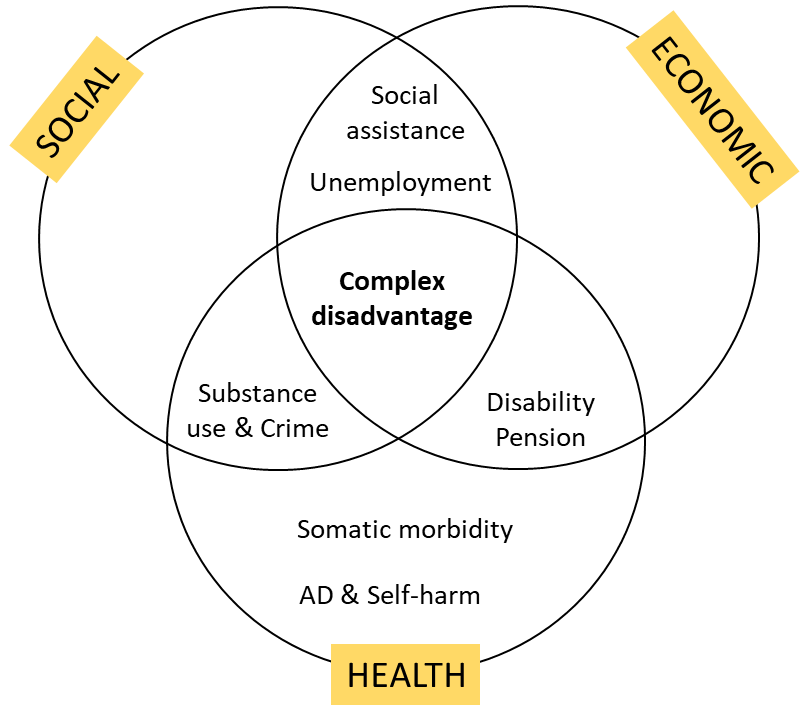


Figure S2: Overview of different outcome dimensions across life domains, considered as individual outcomes and as composite score

Table S1: Overview of ICD-10 diagnosis codes included by indicator

| Indicator | Diagnosis codes | Diagnoses |
| --- | --- | --- |
| AD & Self-harm | F32-F33  F400-F402  F410-F411  F420-F421  F430-F431  X60-X84, Y10-Y34 | Depressive episode, recurrent depressive disorder  Agoraphobia, Social Phobias, Specific Phobias  Panic Disorder, Generalised anxiety disorder  Predominantly obsessional thoughts or ruminations, Predominantly compulsive acts  Acute stress reaction, Post-traumatic stress disorder  Intentional self-harm, Event of undetermined intent |
| Substance use & Crime | F10-F16; F18-F19  G312  G621  I426  K292  K70  K860  O355  R780  T40  T423-T424, T426  T436  T510  T519  X45  X65  Y15  Z503  Z715  Z722  *Crime (any)* | Mental and behavioural disorders due to psychoactive substance use (residual and late-onset psychotic disorder was excluded)  Degeneration of nervous system due to alcohol  Alcoholic polyneuropathy  Alcoholic cardiomyopathy  Alcoholic gastritis  Alcoholic liver disease  Alcohol-induced chronic pancreatitis  Maternal care for (suspected) damage to foetus by drugs  Finding of alcohol in blood  Poisoning by narcotics and psychodysleptics [hallucinogens]  Poisoning by barbiturates, benzodiazepines, other antiepileptic and sedative-hypnotic drugs  Poisoning by psychostimulants with abuse potential  Toxic effect of alcohol – ethanol  Alcohol, unspecified  Accidental poisoning by and exposure to alcohol  Intentional self-poisoning by and exposure to alcohol  Poisoning by and exposure to alcohol, undetermined intent  Drug rehabilitation  Drug abuse counselling and surveillance  Problems related to lifestyle - drug use  *n/a* |
| Somatic morbidity | C  E  I  J | Malignant neoplasms  Endocrine, nutritional and metabolic diseases  Diseases of the circulatory system  Diseases of the respiratory system |
| *Unemployment* | *n/a* | *n/a* |
| *Social assistance* | *n/a* | *n/a* |
| *Early retirement* | *n/a* | *n/a* |
|  |  |  |

Table S2a: Patterns of missingness (n ( %)) by year and indicator after censoring for death and migration in the full sample (total n=13,692)

|  | Unemployment | Social Welfare Receipt | Disability Pension | AD & Self-Harm | Substance Use & Crime | Somatic Morbidity |
| --- | --- | --- | --- | --- | --- | --- |
| 2001 | 176 (1.29) | 176 (1.29) | 176 (1.29) | 85 (0.62) | 104 (0.76) | 85 (0.62) |
| 2002 | 203 (1.48) | 203 (1.48) | 203 (1.48) | 130 (0.95) | 142 (1.04) | 130 (0.95) |
| 2003 | 246 (1.80) | 246 (1.80) | 246 (1.80) | 160 (1.17) | 170 (1.24) | 160 (1.17) |
| 2004 | 303 (2.21) | 303 (2.21) | 303 (2.21) | 206 (1.50) | 228 (1.67) | 206 (1.50) |
| 2005 | 338 (2.47) | 338 (2.47) | 338 (2.47) | 258 (1.88) | 278 (2.03) | 258 (1.88) |
| 2006 | 380 (2.78) | 380 (2.78) | 380 (2.78) | 296 (2.16) | 311 (2.27) | 296 (2.16) |
| 2007 | 428 (3.13) | 428 (3.13) | 428 (3.13) | 342 (2.50) | 362 (2.64) | 342 (2.50) |
| 2008 | 466 (3.40) | 466 (3.40) | 466 (3.40) | 391 (2.86) | 407 (2.97) | 391 (2.86) |
| 2009 | 527 (3.85) | 527 (3.85) | 527 (3.85) | 435 (3.18) | 467 (3.41) | 435 (3.18) |
| 2010 | 577 (4.21) | 577 (4.21) | 577 (4.21) | 494 (3.61) | 523 (3.82) | 494 (3.61) |
| 2011 | 636 (4.65) | 636 (4.65) | 636 (4.65) | 546 (3.99) | 570 (4.16) | 546 (3.99) |
| 2012 | 692 (5.05) | 692 (5.05) | 692 (5.05) | 606 (4.43) | 639 (4.67) | 606 (4.43) |
| 2013 | 753 (5.50) | 753 (5.50) | 753 (5.50) | 661 (4.83) | 688 (5.02) | 661 (4.83) |
| 2014 | 829 (6.05) | 829 (6.05) | 829 (6.05) | 718 (5.24) | 752 (5.49) | 718 (5.24) |
| 2015 | 913 (6.67) | 913 (6.67) | 913 (6.67) | 804 (5.87) | 839 (6.13) | 804 (5.87) |

Table S2b: Patterns of missingness (n ( %)) by year and indicator after censoring for death and migration in individuals with experience of OHC (n=929)

|  | Unemployment | Social Welfare Receipt | Disability Pension | AD & Self-Harm | Substance Use & Crime | Somatic Morbidity |
| --- | --- | --- | --- | --- | --- | --- |
| 2001 | 19 (2.05) | 19 (2.05) | 19 (2.05) | 5 (0.54) | 7 (0.75) | 5 (0.54) |
| 2002 | 19 (2.05) | 19 (2.05) | 19 (2.05) | 13 (1.40) | 14 (1.51) | 13 (1.40) |
| 2003 | 24 (2.58) | 24 (2.58) | 24 (2.58) | 14 (1.51) | 15 (1.61) | 14 (1.51) |
| 2004 | 31 (3.34) | 31 (3.34) | 31 (3.34) | 18 (1.94) | 19 (2.05) | 18 (1.94) |
| 2005 | 35 (3.77) | 35 (3.77) | 35 (3.77) | 26 (2.80) | 28 (3.01) | 26 (2.80) |
| 2006 | 39 (4.20) | 39 (4.20) | 39 (4.20) | 31 (3.34) | 31 (3.34) | 31 (3.34) |
| 2007 | 40 (4.31) | 40 (4.31) | 40 (4.31) | 36 (3.88) | 36 (3.88) | 36 (3.88) |
| 2008 | 49 (5.27) | 49 (5.27) | 49 (5.27) | 39 (4.20) | 45 (4.84) | 39 (4.20) |
| 2009 | 52 (5.60) | 52 (5.60) | 52 (5.60) | 47 (5.06) | 52 (5.60) | 47 (5.06) |
| 2010 | 57 (6.14) | 57 (6.14) | 57 (6.14) | 52 (5.60) | 55 (5.92) | 52 (5.60) |
| 2011 | 61 (6.57) | 61 (6.57) | 61 (6.57) | 56 (6.03) | 56 (6.03) | 56 (6.03) |
| 2012 | 65 (7.00) | 65 (7.00) | 65 (7.00) | 59 (6.35) | 61 (6.57) | 59 (6.35) |
| 2013 | 72 (7.75) | 72 (7.75) | 72 (7.75) | 63 (6.78) | 66 (7.10) | 63 (6.78) |
| 2014 | 84 (9.04) | 84 (9.04) | 84 (9.04) | 70 (7.53) | 79 (8.50) | 70 (7.53) |
| 2015 | 88 (9.47) | 88 (9.47) | 88 (9.47) | 83 (8.93) | 84 (9.04) | 83 (8.93) |

Table S3a: Overlap between placement reasons by time period/ age group

|  | Period I (age 0-6) | | Period II (age 7-12) | | Period III (age 13-19) | |
| --- | --- | --- | --- | --- | --- | --- |
|  | **OHC (behaviour)**** | | **OHC (behaviour)**** | | **OHC (behaviour)**** | |
| **OHC (family)*** | No | Yes | No | Yes | No | Yes |
| **No** | 12,926 | 0 | 13,426 | 45 | 13,223 | 348 |
| **Yes** | 766 | 0 | 213 | 8 | 105 | 16 |
| **Total** | 13,692 | 0 | 13,639 | 53 | 13,328 | 364 |

*Placements for family reasons; **Placements for behavioural reasons (delinquent behaviour)

Table 3b: Frequencies of placement length categories (placement due to family reasons) by gender and time period/age group

|  | Period I (age 0-6) | | Period II (age 7-12) | | Period III (age 13-19) | |
| --- | --- | --- | --- | --- | --- | --- |
| **Placement length** | **Boys** | **Girls** | **Boys** | **Girls** | **Boys** | **Girls** |
| **No placement** | 6,557 | 6,369 | 6,837 | 6,634 | 6,898 | 6,673 |
| **<12 months** | 355 | 327 | 83 | 65 | 25 | 21 |
| **≥12 months** | 47 | 37 | 39 | 34 | 36 | 39 |

*For none of the time periods were the frequency distributions in placement length statistically significant by gender according to chi-squared tests; where placement length was not defined or we could not be sure that placement was ≥12 months, the observation was assigned to the <12 months category

Note: Information on OHC was not available by year, but only by time period. Individuals solely placed due to own behaviour were included in the comparison group for the ordinal regression analysis, and were omitted from the trajectory group analysis.

We also tested for gender differences in type of placement (foster home vs institution; results not reported), and there were no statistically significant differences apart from in Period I (age 0-6), where more boys (5.17%) than girls (4.38%) were placed in institutions.

Table S4: Frequency and prevalence (n (%)) of disadvantage sum-score by experience of OHC and gender.

|  | No OHC experience | | | | | OHC experience | | | | |
| --- | --- | --- | --- | --- | --- | --- | --- | --- | --- | --- |
| Disadvantage sum-score | Men | | Women | | | Men | | Women | | |
|  | n | % | n | % | p-value | n | % | n | % | p-value |
| 0 | 1,873 | 31.9 | 1,765 | 30.0 | 0.007 | 101 | 23.9 | 80 | 20.0 | 0.57 |
| 1 | 2,348 | 40.0 | 2,322 | 39.4 |  | 148 | 35.0 | 143 | 35.8 |  |
| 2 | 1,003 | 17.1 | 1,124 | 19.1 |  | 82 | 19.4 | 91 | 22.8 |  |
| 3 | 397 | 6.8 | 448 | 7.6 |  | 48 | 11.3 | 49 | 12.3 |  |
| 4 | 179 | 2.9 | 168 | 2.9 |  | 24 | 5.7 | 24 | 6.0 |  |
| ≥ 5 | 77 | 1.3 | 59 | 1.0 |  | 20 | 4.7 | 13 | 3.3 |  |
| Total | 5,877 |  | 5,886 |  |  | 423 |  | 400 |  |  |

* individuals with complete follow-up data (n=12,586); p-values derived from Pearson's chi-squared tests for within CA group comparisons between male and female

Table S5: Ordinal regression of OHC and gender on disadvantage sum-score (n=12,586)

|  | Disadvantage sum-score | | | | | | | |
| --- | --- | --- | --- | --- | --- | --- | --- | --- |
|  | Model 1 | | Model 2 | | Model 3 | | Model 4 | |
|  | OR (95% CI) | p-value | OR (95% CI) | p-value | OR (95% CI) | p-value | OR (95% CI) | p-value |
| OHC^a^ | 1.81 (1.59, 2.07) | <0.001 | 1.82 (1.59, 2.07) | <0.001 | 1.70 (1.49, 1.94) | <0.001 | 1.63 (1.35, 1.96) | <0.001 |
| Gender^b^ |  |  | 1.10 (1.03, 1.17) | 0.004 | 1.14 (1.07, 1.21) | <0.001 | 1.13 (1.06, 1.21) | <0.001 |
| Education^c^ |  |  |  |  | 0.57 (0.52, 0.62) | <0.001 | 0.57 (0.52, 0.62) | <0.001 |
| OHC*Sex/gender |  |  |  |  |  | | 1.10 (0.84, 1.42) | 0.50 |

* Model 1: Includes OHC; Model 2: Model 1 + sex/gender; Model 3: Model 2 + education, Model 4: Model 3 + interaction term OHC*gender; ^a^ OHC placement due to family reasons (ref: no placement and placements due to own behaviour); ^b^ women (ref: men); ^c^ highest educational level upper secondary or higher (ref: highest educational level lower than upper secondary); Model 3 and 4 comprise n=12,506 observations due to missing information in the education variable.

Table S6: Model fit criteria

| Polynomial type | BIC* | AIC* | Entropy | Group sizes |
| --- | --- | --- | --- | --- |
| *Intercept* |  |  |  |  |
| 2 groups | -16371.10 | -16339.72 | 0.985 | G1 73.0% / G2 27.0% |
| 3 groups | -15312.80 | -15264.54 | 0.957 | G1 63.1% / G2 21.7% / G3 15.2% |
| 4 groups | -14850.43 | -14785.27 | 0.930 | G1 53.7% / G2 20.8% / G3 18.7% / G4 6.8% |
| 5 groups | -14493.03 | -14410.98 | 0.947 | G1 14.7% / G2 52.9% / G3 8.3% / G4 17.6% / G5 6.4 % |
| *Linear* |  |  |  |  |
| 2 groups | -16148.88 | -16088.55 | 0.985 | G1 73.1% / G2 26.9% |
| 3 groups | -14963.71 | -14872.01 | 0.954 | G1 62.4% / G2 21.8% / G3 15.7% |
| 4 groups | -14553.62 | -14430.54 | 0.950 | G1 58.1% / G2 9.6% / G3 21.7% / G4 10.7% |
| 5 groups | -14148.27 | -13993.82 | 0.942 | G1 56.1% / G2 11.3% / G3 16.4% / G4 6.2% / G5 10.0 % |
| 6 groups | -13885.10 | -13699.27 | 0.938 | G1 8.7% / G2 49.0% / G3 4.6% / G4 16.2% / G5 5.7%/ G6 15.7% |
| *Quadratic* |  |  |  |  |
| 2 groups | -16164.98 | -16075.69 | 0.985 | G1 73.1% / G2 26.9% |
| 3 groups | -14997.46 | -14862.31 | 0.956 | G1 62.3% / G2 21.8% / G3 15.9% |
| 4 groups | -14602.22 | -14421.23 | 0.938 | G1 54.0% / G2 21.1% / G3 18.9% / G4 6.1% |
| 5 groups^a^ | -14203.69 | -13976.84 | 0.949 |  |
| 6 groups | -14027.04 | -13754.34 | 0.926 | G1 21.3% / G2 48.7% / G3 5.6% / G4 13.6% / G5 5.4%/ G6 5.4% |
| *Cubic* |  |  |  |  |
| 2 groups ^a^ | -16195.96 | -16077.71 | 0.986 |  |
| 3 groups | -15036.63 | -14858.05 | 0.955 | G1 62.3% / G2 21.9% / G3 15.8% |
| 4 groups ^a^ | -14660.43 | -14421.52 | 0.938 |  |
| 5 groups ^a^ | -14340.36 | -14041.11 | 0.935 |  |

^a^ Variance matrix nonsymmetric or highly singular.

* BIC = Bayesian Information Criterion; AIC = Akaike Information Criterion; n = 929

The five-group linear polynomial model was chosen as best fitting solution.

Table S7: Group membership probabilities by assigned trajectory group

|  | Mean (SD); min, max | | | | |
| --- | --- | --- | --- | --- | --- |
|  | Member G1  (n=527) | Member G2 (n=100) | Member G3 (n=154) | Member G4 (n=57) | Member G5 (n=91) |
| Probability G1 | 0.97 (0.08);  0.51, 1.00 | 0.06 (0.12);  0.00, 0.49 | 0.00 (0.01);  0.00, 0.08 | 0.00 (0.00);  0.00, 0.01 | 0.02 (0.07);  0.00, 0.47 |
| Probability G2 | 0.02 (0.07);  0.00, 0.48 | 0.92 (0.14);  0.47, 1.00 | 0.00 (0.02);  0.00, 0.24 | 0.00 (0.02);  0.00, 0.12 | 0.01 (0.03);  0.00, 0.29 |
| Probability G3 | 0.00 (0.01);  0.00, 0.16 | 0.00 (0.03);  0.00, 0.26 | 0.97 (0.09);  0.33, 1.00 | 0.03 (0.06);  0.00, 0.28 | 0.00 (0.00);  0.00, 0.00 |
| Probability G4 | 0.00 (0.00);  0.00, 0.06 | 0.00 (0.01);  0.00, 0.06 | 0.02 (0.07);  0.00, 0.42 | 0.97 (0.07);  0.72, 1.00 | 0.00 (0.00);  0.00, 0.00 |
| Probability G5 | 0.00 (0.02);  0.00, 0.27 | 0.02 (0.07);  0.00, 0.39 | 0.00 (0.01);  0.00, 0.17 | 0.00 (0.01);  0.00, 0.08 | 0.97 (0.08);  0.53, 1.00 |

Table S8: Multinomial logistic regression results (n=929); G1 (no disadvantage) as the reference category

|  | Model 1 | | | Model 2 | | | |
| --- | --- | --- | --- | --- | --- | --- | --- |
|  | OR | p-value | 95% CI | | OR | p-value | 95% CI |
| G2 (Health disadvantage)  Women (ref. men) | 0.96 | 0.834 | 0.62, 1.47 | | 0.94 | 0.788 | 0.61, 1.45 |
| Education^a^ |  |  |  | | 1.00 | 0.996 | 0.61, 1.63 |
| G3 (Disabling health disadvantage)  Women (ref. men) | 1.38 | 0.081 | 0.96, 1.98 | | 1.52 | 0.027 | 1.05, 2.20 |
| Education^a^ |  |  |  | | 0.55 | 0.002 | 0.38, 0.81 |
| G4 (Complex disadvantage)  Women (ref. men) | 0.56 | 0.046 | 0.32, 0.99 | | 0.61 | 0.099 | 0.34, 1.10 |
| Education^a^ |  |  |  | | 0.37 | 0.001 | 0.21, 0.65 |
| G5 (Unemployment-related disadvantage)  Women (ref. men) | 0.51 | 0.005 | 0.32, 0.81 | | 0.51 | 0.005 | 0.32, 0.82 |
| Education^a^ |  |  |  | | 0.71 | 0.163 | 0.44, 1.15 |

* Model 1: Includes gender; Model 2: Model 1 + education; ^a^ highest educational level upper secondary or higher (ref: highest educational level lower than upper secondary); Model 2 comprises n=917 observations due to missing information in the education variable.


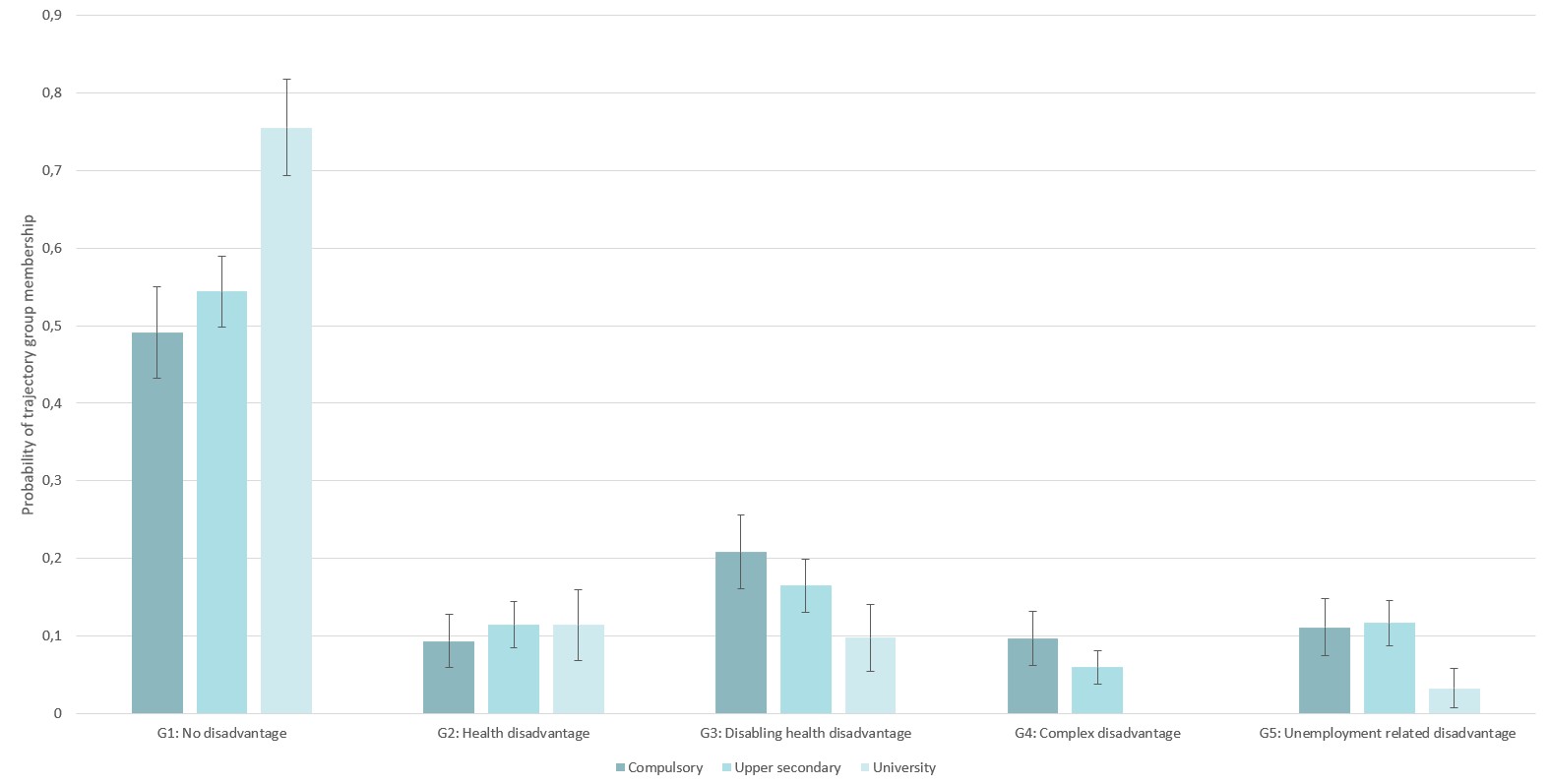


Figure S3: Plot of predicted probabilities of group membership by education, not adjusted for gender.
